# Enhancing the Functionality of a Grid-Connected Photovoltaic System in a Distant Egyptian Region Using an Optimized Dynamic Voltage Restorer: Application of Artificial Rabbits Optimization

**DOI:** 10.3390/s23167146

**Published:** 2023-08-13

**Authors:** Nagwa F. Ibrahim, Abdulaziz Alkuhayli, Abderrahmane Beroual, Usama Khaled, Mohamed Metwally Mahmoud

**Affiliations:** 1 Electrical Department, Faculty of Technology and Education, Suez University, Suez 43533, Egypt; 2 Electrical Engineering Department, College of Engineering, King Saud University, Riyadh 11421, Saudi Arabia; 3 AMPERE Lab UMR CNRS 5005, Ecole Centrale de Lyon, University of Lyon, 36 Avenue Guy de Collongue, 69130 Ecully, France; 4 Department of Electrical Engineering, Faculty of Energy Engineering, Aswan University, Aswan 81528, Egypt

**Keywords:** artificial rabbits optimization (ARO), dynamic voltage restorer (DVR), fault conditions, power quality, photovoltaic (PV)

## Abstract

Photovoltaic (PV) systems are crucial to the production of electricity for a newly established community in Egypt, especially in grid-tied systems. Power quality (PQ) issues appear as a result of PV connection with the power grid (PG). PQ problems cause the PG to experience faults and harmonics, which affect consumers. A series compensator dynamic voltage restorer (DVR) is the most affordable option for resolving the abovementioned PQ problems. To address PQ difficulties, this paper describes a grid-tied PV combined with a DVR that uses a rotating dq reference frame (dqRF) controller. The main goal of this study is to apply and construct an effective PI controller for a DVR to mitigate PQ problems. The artificial rabbits optimization (ARO) is used to obtain the best tune of the PI controller. The obtained results are compared with five optimization techniques (L-SHADE, CMAES, WOA, PSO, and GWO) to show its impact and effectiveness. Additionally, Lyapunov’s function is used to analyze and evaluate the proposed controller stability. Also, a mathematical analysis of the investigated PV, boost converter, and rotating dqRF control is performed. Two fault test scenarios are examined to confirm the efficacy of the suggested control approach. The parameters’ (voltage, current, and power) waveforms for the suggested system are improved, and the system is kept running continuously under fault periods, which improves the performance of the system. Moreover, the findings demonstrate that the presented design successfully keeps the voltage at the required level with low THD% values at the load side according to the IEEE standards and displays a clear enhancement in voltage waveforms. The MATLAB/SIMULINK software is used to confirm the proposed system’s performance.

## 1. Introduction

### 1.1. Motivations

Around the world, every city has distinctive qualities that set it apart from other cities. But the ability of sustainable cities (SCs) to meet present-day demands without jeopardizing the needs of the future is what distinguishes them from communities that exhaust their energies/resources [1,2,3]. While every SC may have something distinctive to offer, it is the ability to balance social, economic, and environmental considerations that allows an SC to endure and prosper over time. SCs are cities whose development plans incorporate sustainable practices to make sure that present demands are satisfied without jeopardizing the capability of future generations to satisfy their own necessities. By doing so, they can achieve a balance that benefits the environment, society, and the economy, providing long-term benefits for the city and its inhabitants [4,5].

With over four billion people living in cities, the urban population (UP) currently makes up over 50 percent of the world’s population. The UP is growing at the expense of the rural population, notably in Asia and Africa, where this number is rising quickly [6,7]. This trend reflects the ongoing process of urbanization, which has become a defining characteristic of the modern era. The concentration of people in urban areas can have significant implications for the environment, economy, and society. As cities grow in size and number, they may face challenges related to infrastructure, transportation, housing, and public services. However, urbanization can also bring benefits such as increased economic opportunities, cultural exchange, and access to essential services. Therefore, it is crucial to develop sustainable and equitable urban policies that address the challenges of urbanization while leveraging its potential benefits. Urban regions are expected to house 70 of every 100 persons globally by the year 2050 [3,6,8].

Cities continue to be desirable locations for talented workers and investors since they contribute significantly to bringing millions of people out of severe poverty and produce more than 80% of the world’s gross domestic product (GDP). As a result, city councils must prioritize the development of regulatory frameworks, infrastructure upgrades, and modern solid waste management to address the increasing concerns surrounding urban growth. These steps are essential to ensuring that cities can efficiently deal with population expansion brought on by both normal growth and rural-to-urban movement [9,10]. The efficient management of these issues is essential for creating livable, sustainable, and thriving urban environments that meet the needs of their inhabitants. In doing so, city councils can create an ecosystem that attracts investment, fosters innovation, and provides access to essential services, thereby contributing to the growth and development of the wider region. Therefore, city councils worldwide must continue to focus on urban development to foster growth, innovation, and prosperity. An SC is a lively urban area that offers ample opportunities while also existing in harmony with the natural environment, ultimately providing a good standard of living for all of its inhabitants [11].

These types of cities prioritize striking a balance between social, economic, and environmental considerations, providing equal opportunities and access to essential services while ensuring that development does not negatively impact the planet’s resources. SCs often prioritize public transportation and green spaces, both of which contribute to healthier living environments for residents. Additionally, SCs also support the utilization of clean energy sources to lessen carbon pollution and advance environmentally beneficial behaviors. An SC is characterized by a cohesive, harmonious, and connected community that prioritizes the clean and efficient use of resources in an interactive, natural environment [12]. New energy policies that rely more on sources of clean energy, such as solar photovoltaic technology (PV), wind, biomass, and fuel cells (FCs), are being implemented by the North African nations. Egypt, among these nations, has a lot of potential for producing green energy (PV and wind sources). Despite this potential, fossil fuels still account for a sizable amount of Egypt’s electrical production. In actuality, 94% of the nation’s overall primary energy consumption comes from fossil fuels (FFs). With Egypt’s small oil and gas reserves, this strong reliance on FFs not only has a negative impact on the environment, but also significantly strains the government’s finances [13,14].

### 1.2. Related Work

The grid-connected PV system has drawn a lot of attention from scholars because of its capacity to produce sustainable and clean energy, especially in rural areas. This system has several advantages, including its accuracy and reliability in generating electricity. The growing electricity demand has made grid-connected PV systems increasingly popular, as they are capable of meeting the energy needs of various sectors [15,16]. Most green sources produce DC power at varying voltage levels, and in order to connect these source outputs to the DC bus in a grid-connected PV system, DC-DC choppers are required [17]. In the meantime, inverters are used to connect AC loads in off-grid mode and the DC bus is connected to the power supply for on-grid applications. However, the operation of the system might be negatively impacted by the switches used in converters [17,18].

The utilization of power electronic devices in hybrid power systems gives rise to operational failures, a range of voltage levels, currents, and abrupt voltage changes, as well as the presence of harmonic components in the current and voltage signals. Poor electrical power quality (PQ) can lead to negative effects such as power outages; overheating of machines, causing reduced lifetimes; machine malfunctions; and damage to sensitive equipment, ultimately resulting in production interruption [19,20].

In a study by the authors of [21,22], a method using a dual voltage source inverter (DVSI) was examined to improve the efficiency of microgrids (µGs). This method allows for a more reliable power supply to sensitive loads within the µG. Meanwhile, the authors of [23] proposed another technique to improve the wind energy system performance by using a dynamic voltage resistor (DVR) and a static transfer switch (STC). The proposed DVR and STC can mitigate the effects of disturbances such as voltage sags ($V_{S}$) and interruptions in power generation within the system. A novel system was suggested in [24], which combines a distributed power flow controller with a proton exchange membrane FC and a Z-source inverter to achieve acceptable performance.

In [25], the authors introduced a DVR system that uses an ultra-capacitor as energy storage to power the VSI and mitigate PQ issues. Meanwhile, the authors of [26] proposed a DVR system for $V_{S}$ problems, which utilizes an ultra-capacitor as well. In [27], it was demonstrated that the use of a DVR in a renewable FC generator system can improve the PQ of the electrical power system. As for three-phase systems, the authors of [28] discussed shunt filters and their control techniques for purposes such as compensating harmonics and reactive power.

In [29], shunt filters are elaborated on as devices that are utilized for reactive power and harmonics compensation. Additionally, the authors of [30] describe a series of active filters with various control techniques and voltage compensation utilized to facilitate the integration of green systems into the grid, both for on–off grid modes. The control strategy that incorporates these devices presents a hurdle, which is that it needs to be simple and applicable. The use of PI controllers, which is a simple and widely used control method, is prevalent in controlling power electronic devices [31]. One of its applications is in controlling the DC chopper for connecting FCs to the electrical grid, while another application is in controlling the STATCOM in wind systems [32].

In hybrid green systems, power electronic components are integrated, causing the entire system to be complicated and nonlinear, which makes it challenging to adjust the PI controller parameters [33,34]. To address this issue, various optimization methods are used to adjust the controller gains. The application of whale optimization method (WOM)-based fractional PI controllers for UPQC and STATCOM in recent power systems that involves nonlinear loads and wind systems was presented in [35]. The obtained results show that the optimized controllers helped in the mitigation of unstable harmonics and voltage. In refs. [36,37], it was shown that utilizing optimized STATCOM controllers helps to achieve the high penetration scenarios of switched reluctance and induction-machine-based wind turbines in modern power grids. A grid-connected PV system employing the adaptive generalized maximum Versoria criterion with PQ improvement and a harmony search-optimizer-based PI controller for connecting FCs to the grid was presented in [38]. In [39], distinct optimization techniques (manta ray foraging method, slap swarm method, WOM, jellyfish search method, grasshopper search method, circle search method, and enhanced transient search method) were used to determine the PEM-FC parameters. Artificial rabbits optimization (ARO) was recommended for the DVR control system because the results of the comparisons between this approach and the previously listed approaches show that it was superior. A comparison with the previous studies that addressed the same research point is made in Table 1 to demonstrate the significance and efficiency of the present study.

### 1.3. Contributions

Through the resolution of a set of 31 benchmark functions and five engineering issues, the efficiency of ARO is evaluated in contrast to that of other popular optimizers. The findings demonstrate that ARO consistently outperformed its examined competitors in terms of resolving engineering issues and benchmark functions. Therefore, the ARO approach is taken into account in our study [48]. The ARO is advised to optimize the DVR controllers in order to obtain a more precise DVR control system. The ARO is based on the rabbits’ natural detour foraging (DF) technique and a random concealment approach [49]. In this research paper, the authors implement and apply the L-SHADE, CMAES, WOA, PSO, GWO, and ARO techniques for the DVR to determine the optimum gains of the controllers. This work aims to improve the performance of an on-grid PV system that is used to supply power to a new city in Egypt (New Suez). To calculate the optimal size of the operated renewable sources, the authors also consider several economic factors, such as net present expenses and electricity prices. Our goal is to enhance the performance of the system using the six studied techniques, which is expected to result in improved system stability, reduced system losses, and enhanced overall system efficiency. When compared to DVR based on any studied optimizer PI controller and without DVR under various conditions, the resulting total harmonic distortion (THD) is reduced with DVR based on the ARO-PI controller, which is a quick transient response for resolving PQ problems. Furthermore, controller stability is investigated to prove the role of the ARO method. Comparisons between the ARO-PI controller and the L-SHADE, CMAES, WOA, PSO, and GWO-PI controllers’ performance in reducing the THD and boosting the system performance are made to further highlight the benefits of the ARO approach. The results are quite inspiring and helpful for these applications.

### 1.4. Paper Organization

There are six parts of this article as follows: The introduction is in Section 1. The description and modeling of the system under consideration are illustrated in Section 2. Section 3 presents a detailed DVR control mechanism. Section 4 presents the applied optimization techniques and investigates the system stability. Section 5 discusses the simulation results and comparisons, and Section 6 offers the conclusion.

## 2. Description and Modeling of the Investigated System

### 2.1. System Description

**Figure 1:**
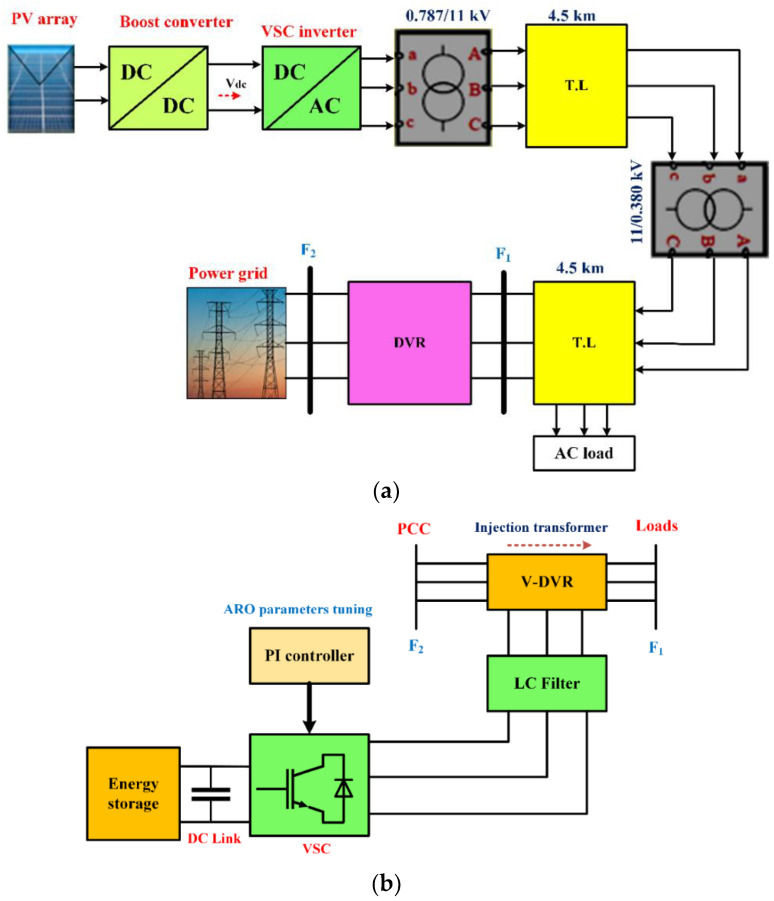
(**a**) Diagram illustrating a PV system incorporating a DVR and (**b**) DVR components.

The planned grid-connected PV system is designed to provide electricity to Suez, a newly developed region in Egypt that is situated in a highly advantageous environment. The investigated system includes a PV generation source connected to the grid, which uses DC-DC converters to produce power. The inverter is utilized to change the output power from DC to AC, and step-up transformers are employed to link the electrical system to Egypt’s intended 11 KV community grid. Step-down transformers are employed to deliver electricity to single and three-phase AC loads in the neighborhood, where the voltage is reduced from 11 kV to 0.380 kV and the frequency is 50 Hz. The electrical system is situated 4.5 km from the 11 kV grid. The proposed system was tested at a source voltage of 0.380 kV, as shown in Figure 1a. To mitigate any disturbances that may affect the system, a DVR is used and includes a storage unit, a VSI, power transformers, and L-C filters, as shown in Figure 1b. The two optimized PI controllers are used with the ARO method to regulate the VSI. The addressed system parameters are listed in Table 2.

### 2.2. PV System Modeling

A parallel arrangement of an equivalent diode structure and a light-dependent current supply commonly serves as the equivalent circuit for a PV. The amount of light that strikes the cell is directly correlated with its current output. The output current eventually goes to zero when the load resistance exceeds a certain point because the PV cell is incapable of sustaining a constant current as the load resistance rises. The PV model accounts for the photocurrent *I_L_*’s temperature dependency, as well as the diode’s saturation current (*I*_0_), the *R_S_* and *R_p_* series and parallel resistance, and so forth. The net current of the cell is determined by the difference between the *I_L_* and the *I*_0_, as given in Equation (1). The PV cell’s modeling and I-V properties are described in the equations below [50].

(1) $$I=I_{L}-I_{o}\left\{e^{\frac{q\left(v+IRS\right)}{nkT}}-1\right\}$$

(2) $$I=I_{L}\left(T_{1}\right)\left(1+k_{0}\left(T-T_{1}\right)\right)$$

(3) $$I_{L}\left(T_{1}\right)=G*\frac{I_{SC\left(T_{1},nom\right)}}{G_{\left(nom\right)\;\;\;\;}}$$

(4) $$k_{0}=\left(I_{SC\left(T2\right)}-I_{SC\left(T1\right)}\right)/\left(T_{2}-T_{1}\right)$$

(5) $$I_{0}=I_{0\left(T1\right)}*{\left(\frac{T}{T_{1}}\right)}^{\frac{3}{n}}*e^{\frac{qvg}{nk}}*\left(\frac{1}{T}-\frac{1}{T_{1}}\right)$$

(6) $$I_{0\left(T1\right)}=\frac{I_{SC\left(T1\right)}}{e^{\frac{qvoc\left(T1\right)}{nkT1}}-1}$$

(7) $$R_{S}=-\frac{dv}{dI_{voc}}-\frac{1}{X_{v}}$$

(8) $$X_{v}=I_{0\left(T1\right)}\;\frac{q}{nkT1}\;e^{\frac{qvoc\left(T1\right)}{nkT1}}$$

The values for the constants used in the above equations are derived from the PV array manufacturer’s specifications.

The symbols $I_{SC}$, $V_{OC}$, *G*, *k*, *q*, *T*, *n*, and $V_{g}$ are the short circuit current, open circuit voltage, irradiance (in W/m^2^), Boltzmann’s constant (1.38 × 10^−23^), electron charge (1.60 × 10^−19^), the temperature in degrees Celsius, ideality factor, and band gap voltage, respectively. The used symbols are fully defined in [51].

The formulas presented here can be used to determine how much power the PV panel is producing at any given moment [52].

(9) $$P_{PV}\left(t\right)=S_{r}\left(t\right)\;a\;\eta$$

The equation for the total output power is given as $P_{PV}$ = $S_{r}$ a *η*, where $P_{PV}$ represents the generated power, *S_r_* is the amount of solar radiation received, a is the surface area, and *η* is the efficiency.

(10) $$P_{t}\left(t\right)=N_{PV}\;P_{PV}\left(t\right)$$

where $P_{t}$ represents the total power output and $N_{PV}$ is the number of employed PV panels.

## 3. DVR Control System and Applications

### 3.1. DVR Operation and Control

In this section, the application of the investigated DVR for enhancing the PQ in modern power systems is covered. As shown in Figure 1b, the DVR is made up of a number of parts, including a storage unit, a VSI, an LC filter, and a transformer. The VSI receives power from the energy storage and uses an injection transformer to provide a sufficient voltage to restore the load voltage ($V_{L}$). However, due to the presence of some harmonics in the VSI output, caused by the IGBT switches, an LC filter is employed to alleviate this issue [53].

**Figure 2:**
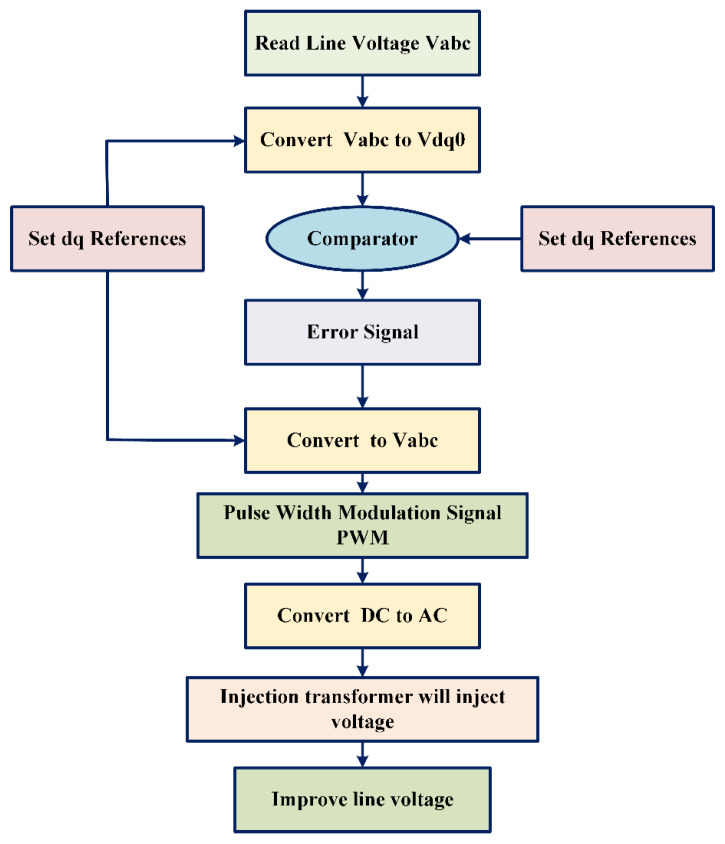
Flowchart operation of DVR.

The DVR senses any disturbances at feeder F_2_ and applies the necessary voltage via a feeding transformer to lessen them. After treating the disturbance, the DVR emits a sinusoidal and balanced wave that is clean of all distortions and side effects that the disturbance may have had on the system. The DVR’s operational flowchart is shown in Figure 2, which gives a visual picture of how the DVR reacts to power supply interruptions. Overall, the suggested DVR design is a thorough and practical approach to enhancing the PQ in the recent power system.

### 3.2. Investigated DVR Control Scheme

**Figure 3:**
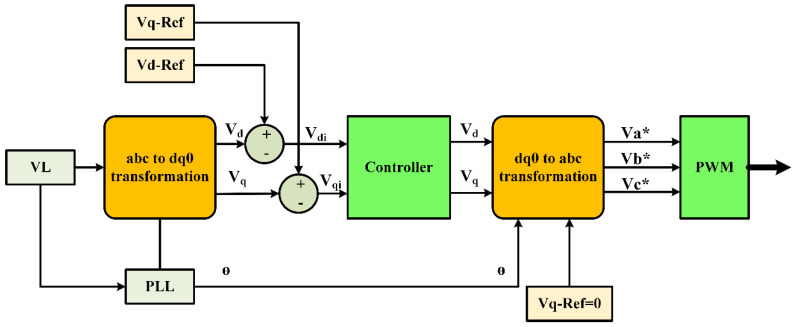
Control block diagram of the DVR.

The crucial function of the controller in the DVR system is discussed in this section. As detailed in earlier work, a closed-loop control system is employed in a rotating dq reference frame to retain control over the DVR system [54]. The DVR controller outputs an appropriate pulse into the VSI’s IGBT switch when abnormalities take place. Figure 3 shows a schematic of the DVR’s control system. Through the use of the given equations, the three-phase coordinate system is transformed into the dq0 coordinate system. This controller is a vital part of the DVR system because it allows for the system to adapt to disruptions rapidly and efficiently and to maintain a high-quality power supply.

(11) $$V_{d}=\frac{2}{3}\left[V_{a}sin\omega t+V_{b}\sin\left(\omega t-\frac{2\pi }{3}\right)+V_{c}sin\left(\omega t+\frac{2\pi }{3}\right)\right]$$

(12) $$V_{q}=\frac{2}{3}\left[V_{a}cos\omega t+V_{b}\cos\left(\omega t-\frac{2\pi }{3}\right)+V_{c}cos\left(\omega t+\frac{2\pi }{3}\right)\right]$$

(13) $$V_{0}=\frac{1}{3}\left[V_{a}+V_{b}+V_{c}\right]$$

This section explains how to calculate the disruption in the dq coordinate frame. The desired value is transformed back to the ABC coordinate framework and the dq coordinate system is compared to it. In addition, a phase-locked loop (PLL) is used to gauge the system’s frequency. The provided equations illustrate how the difference between the dq voltage’s actual and standard values is used as an input to the PI controller of the DVR. The PLL has a serious role in ensuring that the frequency of the system is accurately measured, which is necessary for the accurate calculation of the disturbance in the dq coordinate system. The PI controller uses the error in the dq voltage to control the output of the DVR and mitigate any disturbances, thus ensuring that the system remains stable and supplies a high PQ. This process highlights the importance of precise measurement and control in maintaining a robust and reliable power system.

(14) $$error-d\left(t\right)=Vd_{Ref}-Vd$$

(15) $$error-q\left(t\right)=Vq_{Ref}-Vq$$

**Figure 4:**
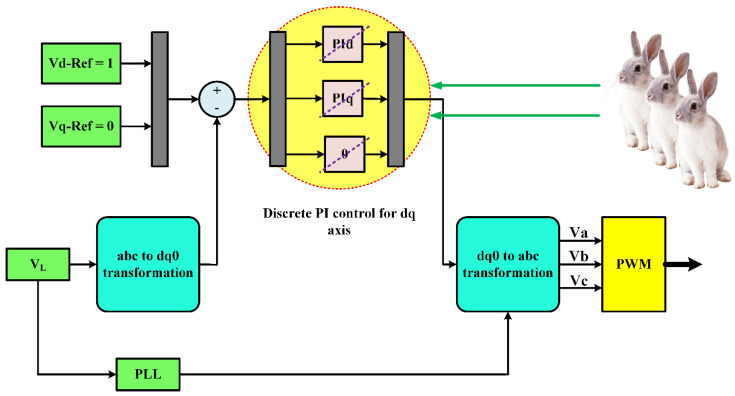
PI control circuit of the DVR.

The error-d signal is received by the d-axis PI controller, whereas the error-q signal is received by the q-axis PI controller. The MATLAB Simulink simulation of the PI controller’s control circuit is shown in Figure 4. In order to create the proper IGBT pulses for the VSI, the output is then transformed to ABC coordinates and sent to the PWM. The deviation between the dq voltages and the standard voltages of the dq coordinates forms the input of the PI controller. The three-phase $V_{L}$ is monitored and translated to dq0 coordinates. While the standard voltage for q is 0, the reference voltage for d is set to 1 p.u. (rated voltage). The d and q error signals are handled by two controllers, $PI_{d}$ and $PI_{q}$, respectively. When the PI controller generates an output, it converts it to ABC and sends it to the PWM to activate the VSI’s IGBT. Due to the nonlinear uncertainties and restrictions present, such as temperature and irradiation, tuning the PI controllers is difficult. Such complex problems can be beyond the capabilities of conventional optimization techniques. Nevertheless, these problems can be solved by using artificial intelligence techniques like VBRL. One of the most effective ways to fine-tune the PI control parameters is to use the ARO and GWO methods since they can react quickly to changing circumstances and guarantee global convergence. Consequently, ARO is a fantastic remedy for these issues.

## 4. Applied Optimization Methods and Stability Analysis

### 4.1. GWO Technique

**Figure 5:**
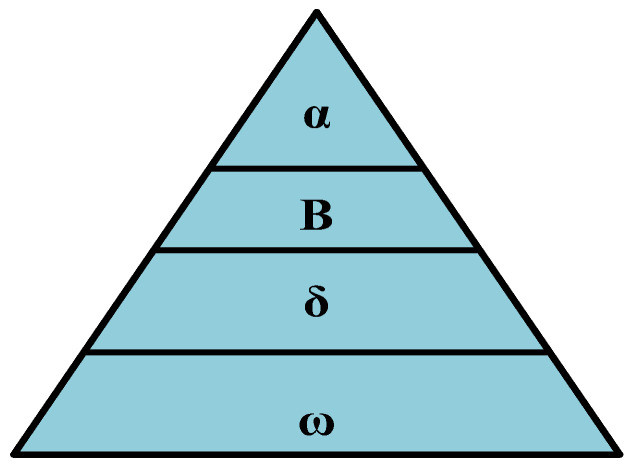
The social structure of GWs.

This technique is a meta-heuristic algorithm and was introduced in [55]. It mimics the social organization and hunting methods of wild grey wolves (GWs). Four different simulation types (Alpha (α), Beta (β), Delta (δ), and Omega (ω)) are used in the GW hierarchy, as shown in Figure 5. The role and behavior towards the prey of the four simulation types were presented in [55]. The β and δ GWS are rated as the second- and third-best options, respectively, and the α GW is regarded as the finest fit. The remaining solutions are categorized as ω and are thought to be the least significant. The three phases of hunting behavior are tracking, chasing, and approaching the prey, surrounding and agitating the prey until it ceases to move, and finally, killing the animal being hunted.

Equations (16) and (17) are used to model the encircling behavior, where t represents the present iteration, $A^{\to }$ and $C^{\to }$ are the vectors of coefficients, and $XP^{\to }$(*t*) reflects the victim’s location vector, while $X^{\to }$ represents the place vector of a GW. The coefficients $A^{\to }$ and $C^{\to }$ are calculated in (18) and (19) as follows:

(16) $$D^{\to }=\left|C\;XP^{\to }\left(t\right)-X^{\to }\left(t\right)\right|$$

(17) $$X^{\to }\left(t+1\right)=XP^{\to }\left(t\right)-A^{\to }D^{\to }$$

(18) $$A^{\to }=2a^{\to }r1^{\to }-a^{\to }$$

(19) $$C^{\to }=2r2^{\to }$$

where $a^{\to }$ is linearly reduced from 2 to 0 all over iterations, and $r1^{\to }$ and $r2^{\to }$ are arbitrary vectors in the variety [0, 1].

The first three optimal solutions are saved using this procedure, and all other search agents, including the Omegas, are needed to adjust their placements by the places of the best search agents. The following formulas are suggested to accomplish this:

(20) $$Da^{\to }=\left|C1^{\to }Xa^{\to }-X^{\to }\right|,\;DB^{\to }=\left|C2^{\to }XB^{\to }-X^{\to }\right|,\;D\delta^{\to }=\left|C3^{\to }X\delta^{\to }-X^{\to }\right|$$

(21) $$X1^{\to }=Xa^{\to }-A1{\left(Da^{\to }\right)}^{\to },\;X2^{\to }=XB^{\to }-A2{\left(DB^{\to }\right)}^{\to },\;X3^{\to }=X\delta^{\to }-A3{\left(D\delta^{\to }\right)}^{\to }$$

(22) $$X^{\to }\left(t+1\right)=\frac{X1^{\to }+X2^{\to }+X3^{\to }}{3}$$

**Figure 6:**
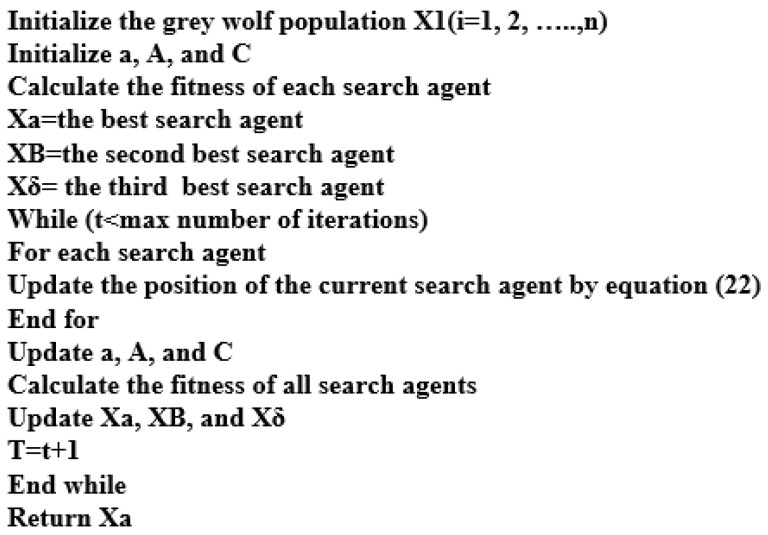
The code outlines the steps of the GWO algorithm [56].

The GWO’s pseudo-code is shown in Figure 6. The tracking, circling, and attacking of prey, as reported in [56], are used to mathematically model the social hierarchy. The GWO algorithm’s maximum iteration and number of search agents are 100 and 30, respectively.

### 4.2. ARO Technique

**Figure 7:**
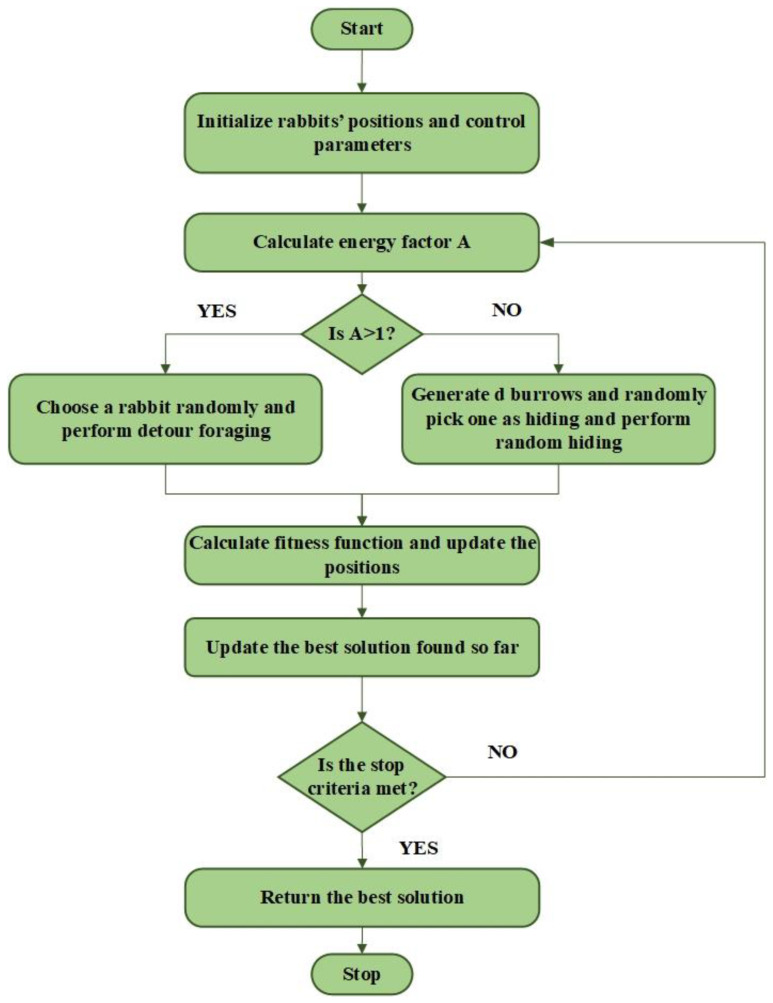
ARO flowchart.

The ARO technique is influenced by the natural survival techniques employed by rabbits. Rabbits use a method of foraging known as diversion to find feed away from their homes. They dig burrows around their nests to hide from hunters and other predators, and depending on the situation, they may opt to undertake either diversion foraging or arbitrary hiding on their vigor [48]. Whenever they have enough energy, they will travel great distances from their homes in search of food, and if they are low on energy, they will haphazardly hide in the nearby tunnels. The procedures and equations used to update the rabbits’ places are detailed in the next parts and are summarized in Figure 7 [48].

(a). Alternate between exploration and exploitation

The energy shrink is represented by this stage. Rabbits can select between haphazard concealment and detour foraging. This relies on the amount of energy a rabbit has; thus, to imitate which one it will choose, an energy factor *A*(*t*) is derived using Equation (23). A rabbit will engage in random concealment when A(t) > 1, and DF when A(t) ≤ 1.

(23) $$A\left(t\right)=4\left(1-\frac{t}{T}\right)Ln\frac{1}{r}\;\;\;\;\;\;\;\;\;\;\;\;\;\;\;\;$$

where *r* is selected at random from the range of (0, 1).

(b). Exploration

The second stage represents the DF. To keep predators away from their homes, rabbits forage for food outside of their homes. According to Equation (24), rabbits look for food at random based on where one is in relation to the other.

(24) $$\vec{P_{i}}\left(t+1\right)=\vec{X_{j}}\left(t\right)+R\left(\vec{X_{i}}\left(t\right)-\vec{X_{j}}\left(t\right)\right)+round\left(\frac{1}{2}\;\left(\frac{5}{100}+r_{1}\right)\right)n_{1},i,\;j=1,\ldots ,n\;and\;i\neq j$$

(25) R=L C

(26) $$L=\left(e-e^{{\left(\frac{t-1}{T}\right)}^{2}}\sin\left(2\pi r_{2}\right)\right)$$

(27) $$c\left(k\right)=\{\begin{array}{c} 1\;\;\;\;\;\;\;\;if\;k=g\left(t\right)\\ 0\;\;\;\;\;\;\;\;\;\;\;\;\;\;\;\;\;\;\;\;\;\;\;else \end{array}k=1,\ldots ,\left[r_{3}\;.d\right]$$

(28) g=rand perm(d)

(29) $$n_{1}\sim N\left(0,1\right)$$

where $\vec{P_{i}}\left(t+1\right)$, $\vec{X_{i}}\left(t\right)$, n, d, T, L, ($r_{1}$, $r_{2}$, $r_{3}$), $n_{1}$, *c*, and *R* are the applicant’s place of *i*th rabbit at *t* + 1, the *i*th rabbit’s location at t, the bunny rabbit population size, the number of variables, the maximal number of iterations, the crusade leap of rabbits, three random numbers between (0, 1), the variable that is subject to the standard normal distribution, the mapping vector, and the sprinting operator that mimics the gait of rabbits, respectively.

(c). Exploitation

Random hiding is represented by this stage. To choose a random tunnel to cover in and flee from predators, every rabbit has d holes around its home. Equation (30) creates these holes for every rabbit.

(30) $$\vec{b_{i,j}}\left(t\right)=\vec{X_{i}}\left(t\right)+H\;g\;\vec{X_{i}}\left(t\right),\;i=1,\;n\;and\;j=1,\;\ldots ,\;d$$

(31) $$\;\;\;\;H=\frac{T-t+1}{T}\;r_{4}$$

(32) $$g\left(k\right)=\{\begin{array}{c} 1\;\;\;\;\;\;\;\;if\;k=j\\ 0\;\;\;\;\;\;\;\;\;\;\;\;\;\;else \end{array}k=1,\ldots ,d$$

(33) $$\vec{P_{i}}\left(t+1\right)=\vec{X_{i}}\left(t\right)+R\left(\;r_{4}\;\vec{b_{i,r}}\left(t\right)-\vec{X_{i}}\left(t\right)\right)\;i=1,\;\ldots ,n$$

(34) $$g_{r}\left(k\right)=\{\begin{array}{c} 1\;\;\;\;\;\;\;\;if\;k=\left[r_{5}\;d\right]\\ 0\;\;\;\;\;\;\;\;\;\;\;\;\;\;\;\;\;\;\;\;\;\;\;else \end{array}k=1,\ldots ,d$$

(35) $$\vec{b_{i,r}}\left(t\right)=\vec{X_{i}}\left(t\right)+H\;g_{r}\;\vec{X_{i}}\left(t\right),\;i=1,\;\ldots ,\;n$$

(36) $$\vec{X_{i}}\left(t+1\right)=\{\begin{array}{c} \vec{X_{i}}\left(t\right)\;f\left(\vec{X_{i}}\left(t\right)\right)\;\leq \;f\left(\;\vec{P_{i}}\left(t+1\right)\right)\;\;\;\;\;\;\\ \vec{P_{i}}\left(t+1\right)\;\;f\left(\vec{X_{i}}\left(t\right)\right)>\;f\left(\;\vec{P_{i}}\left(t+1\right)\right) \end{array}$$

where H, $\vec{b_{i,j}}$, $\vec{b_{i,r}}$, and ($r_{4},\;r_{5}$) are the hiding parameter; the *j*th burrow for the *i*th rabbit; the randomly selected burrow for hiding for the *i*th rabbit, as exposed in Equation (35); and the arbitrary numbers flanked by (0, 1), respectively.

According to Equation (33), the *i*th a rabbit will make an effort to alter its place in accordance with the randomly selected burrow. Ultimately, if the suitability of the candidate’s location of the *i*th rabbit is larger than that of the preceding one, as given by Equation (36), the rabbit will depart its present place and stay at the candidate’s spot after either DF or random hiding.

### 4.3. Optimal Controller Design using Different Optimization Methods

This part of the research study focuses on tuning the parameters of two PI controllers that are tasked with operating a DVR by applying the L-SHADE, CMAES, WOA, PSO, GWO, and ARO (proposed) methods. The goal is to test how a controlled DVR affects a green source’s voltage, current, and power waveforms, while also ensuring that the renewable source continues to operate under unusual operating circumstances like a three-phase fault, voltage sag, and swell. To find the two PI controller parameters’ optimum values with the lowest integral square error (ISE), as seen In Equation (37), the ARO and GWO approaches are used. Both algorithms are used to control the inserted voltage from the DVR to the system in the event of a three-phase fault to reduce the error between the standard and real voltage values in dq coordinates. The two PI controllers’ optimized parameters with six different optimizers are provided in Table 3, and the later sections will explore how they affect the system performance under abnormal operating conditions.

(37) $$\mathrm{ISE}=\int_{0}^{\infty }e^{2}\left(t\right)dt$$

### 4.4. Analysis of the Investigated System’s Stability

The stability analysis (SA) was evaluated using a variety of concepts, involving the Bode diagram, zero pole mapping, and Lyapunov function (LF) [57]. The LF is taken into account since it was proven to be effective in solving a number of engineering problems [58]. Based on $V_{DVR}=V_{L}-V_{grid}\;$, with the proposed controller, the system’s standard voltage, or low pass filter (LPF), will be detected, whereas all upsets and uncertainties are calculated and instantly corrected. An LPF represented as $G_{\nu }\left(s\right)$ is used in this study to make the recommended controller design easier. $G_{\nu }\left(s\right)$ was, in fact, selected as a first-order filter.

$$G_{\nu }\left(s\right)=\frac{1}{\left(1+\tau_{\nu }\right)}.$$

$V_{L}$ is written as follows:

(38) $$V_{L}=L^{-1}\left\{\frac{1}{1+\tau_{\nu }}\right\}*\left(V_{grid}+V_{DVR}\right)$$

where the symbols $\tau_{\nu }$, $L^{-1}$, and ∗ are the time constant, inverse Laplace transformation, and convolution operator, respectively.

Equation (38) represents the following dynamics:

(39) $${\dot{V}}_{L}=\frac{V_{DVR}}{\tau_{\nu }}+\Delta_{\nu }$$

where $\Delta_{\nu }=-\frac{V_{L}}{\tau_{\nu }}+\frac{V_{grid}}{\tau_{\nu }}$ and the symbol $\Delta_{\nu }$ represent the lumped uncertain term.

**Figure 8:**
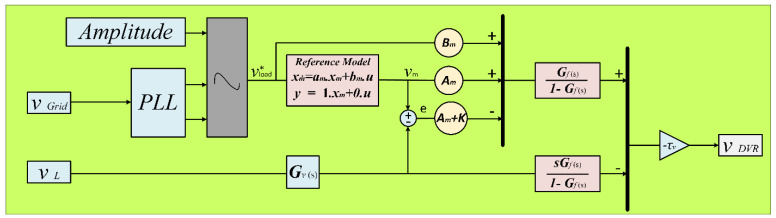
Proposed DVR control configuration for investigating SA.

The recommended DVR control system’s organizational structure is depicted in Figure 8. The controller generates a standard $V_{L}$ with a typical amplitude, and three PLLs are used to synchronize the frequency and phase with the $V_{grid}$ [59]. The investigated controller has two inputs ($V_{L}$ and $V_{grid}$) and a single output ($V_{DVR}$ reference). The inputs are a part of the voltage control loop. The PWM, which generates the switching signals for the converter, is driven by the output. The explored control technique should be combined with a trustworthy linear reference model to guarantee that the closed-loop structure reacts as required [60]. The following equation is written as the $V_{DVR}$ control signal as follows:

(40) $$V_{DVR}=\tau_{\nu }\;\left(A_{m}\;\nu_{m}+B_{m}V^{*}{}_{L}\right)-\tau_{\nu }\left(A_{m}+K\right)e\left(t\right)-\tau_{\nu }\;\left[L^{-1}\left\{G_{f}\left(t\right)\right\}*\left({\dot{V}}_{L}-\frac{V_{DVR}\;}{\tau_{\nu }}\right)\;\right]\;$$

The procedure drawn in [61] allows for the evaluation of the LF bounds for $V_{L}$. The SA is executed using the LF below.

(41) $$V\left(t\right)=V_{L}\;V^{T}{}_{L}$$

With the derivation of (41) and the prearranged $V_{L}$ dynamics (39) and $V_{DVR}$ (40), the SA is performed as follows:

$$\begin{array}{ll} \dot{V}\left(t\right)=\dot{{\dot{V}}_{L}{}^{T}V_{L}+V_{L}{}^{T}{\dot{V}}_{L}}= & \left(\frac{V_{DVR}{\;}^{T}}{\tau_{\nu }}+\Delta_{\nu }{}^{T}\right)V_{L}+V_{L}{}^{T}\left(\frac{V_{DVR}\;}{\tau_{\nu }}+\Delta_{\nu }\right)\\ & =V_{L}\left\{V_{m}{}^{T}A_{m}{}^{T}+{\left(V^{*}{}_{L}\right)}^{T}B_{m}{}^{T}\right\}-V_{L}\left\{\left(V_{m}{}^{T}-V_{L}{}^{T}\right)\left(A_{m}{}^{T}+K^{T}\right)\right\}\\ & +V_{L}\left\{\Delta_{\nu }{}^{T}-L^{-1}\left\{G_{f}\left(t\right)\right\}*\left(\Delta_{\nu }{}^{T}\right)\right\}+V_{L}{}^{T}\left\{A_{m}V_{m}+B_{m}V^{*}{}_{L}\right\}\\ & -V_{L}{}^{T}\left\{\left(A_{m}+K\right)\left(V_{m}-V_{L}\right)\right\}+V_{L}{}^{T}\left\{\Delta_{\nu }-L^{-1}\left\{G_{f}\left(t\right)\right\}*\left(\Delta_{\nu }\right)\right\}\\ & =V_{L}\left\{{\left(V^{*}{}_{L}\right)}^{T}B_{m}{}^{T}-V_{m}{}^{T}K^{T}\right\}+V_{L}\left\{V_{L}{}^{T}\left(A_{m}{}^{T}+K^{T}\right)\right\}\\ & +V_{L}\left\{L^{-1}\left\{1-G_{f}\left(t\right)\right\}*\left(\Delta_{\nu }{}^{T}\right)\right\}+V_{L}{}^{T}\left\{B_{m}V^{*}{}_{L}-KV_{m}+\left(A_{m}+K\right)V_{L}\right\}\\ & +V_{L}{}^{T}\left\{L^{-1}\left\{1-G_{f}\left(t\right)\right\}*\left(\Delta_{\nu }\right)\right\} \end{array}$$

$$\dot{V}\left(t\right)=V_{L}{}^{T}\left(A_{m}{}^{T}+K^{T}+A_{m}+K\right)V_{L}+{\left(V^{*}{}_{L}\right)}^{T}B_{m}{}^{T}V_{L}+V_{L}{}^{T}B_{m}V^{*}{}_{L}-V_{m}{}^{T}K^{T}V_{L}-\;\;V_{L}{}^{T}KV_{m}+L^{-1}\left\{1-G_{f}\left(t\right)\right\}*\left(\Delta_{\nu }{}^{T}V_{L}+V_{L}{}^{T}\Delta_{\nu }\right)$$

When $Q=A_{m}{}^{T}+K^{T}+A_{m}+K$, the equation is expressed as follows:

$$\dot{V}\left(t\right)\leq \lambda_{max}\left(Q\right)\|V_{L}{\|}^{2}+2\|B_{m}V^{*}{}_{L}\|\|V_{L}\|+2\|KV_{m}\|\|V_{L}\|+L^{-1}\left\{1-G_{f}\left(t\right)\right\}*\left(\frac{2\|V_{L}\|{}^{2}}{\tau_{\nu }}\right)+L^{-1}\left\{1-G_{f}\left(t\right)\right\}*\left(\frac{2\|V_{L}\|\|V_{grid}\|}{\tau_{\nu }}\right)\;=L^{-1}\left\{\lambda_{max}\left(Q\right)+\frac{2}{\tau_{\nu }}-\frac{2}{\tau_{\nu }}G_{f}\left(t\right)\right\}*\|V_{L}\|{}^{2}+2\|B_{m}V^{*}{}_{L}\|\|V_{L}\|+2\|KV_{m}\|\|V_{L}\|+L^{-1}\left\{1-G_{f}\left(t\right)\right\}*(\frac{2\|V_{L}\|\|V_{grid}\|}{\tau_{\nu }}$$

where *Q* is the non-positive value that is semi-fixed with the Hurwitz matrix $(A_{m}+K),\lambda_{max}\left(Q\right)<zero$, and is the supreme eigenvalue of *Q*. $\zeta =2\|B_{m}V^{*}{}_{L}\|+2\|KV_{m}\|+L^{-1}\left\{1-G_{f}\left(t\right)\right\}*\left(\frac{2\|V_{grid}\|}{\tau_{\nu }}\right)$ is a limited signal with the upper limit of P, where p is a positive number.

(42) $$\dot{V}\left(t\right)\leq (\lambda_{max}\left(Q\right)+\frac{2}{\tau_{\nu }})\|V_{L}{\|}^{2}+2\zeta \|V_{L}\|$$

When Young’s inequality is functional,

(43) $$\dot{V}\left(t\right)\leq [\lambda_{max}\left(Q\right)+\frac{2}{\tau_{\nu }}+\varepsilon^{2}]\|V_{L}{\|}^{2}+\frac{\zeta^{2}}{\varepsilon^{2}}\;\leq -\lambda_{1}V\left(t\right)+\lambda_{2}$$

where $\lambda_{1}=[\lambda_{max}\left(Q\right)+\frac{2}{\tau_{\nu }}+\varepsilon^{2}],\;\lambda_{2}=\frac{p}{\varepsilon^{2}}$, and is a tuning coefficient to compute the $\lambda_{2}$ size.

$\lambda_{1}>zero$ yields the precise design for the error signal feedback gain ($A_{m}+K$). So, (44) is written as follows:

(44) $$0\leq V\left(t\right)\leq V\left(0\right)e^{-\lambda_{1}t}+\frac{\lambda_{2}}{\lambda_{1}}\left(1-e^{-\lambda_{1}t}\right)$$

When t→∞, $e^{-\lambda_{1}t}$ tends to be 0, so V(t) in (28) has an upper limit of $\frac{\lambda_{2}}{\lambda_{1}}$. As a result, for all t≥0, V(t) has no upper or lower bounds. The work mentioned above shows that the closed-loop structure is robust and stable with regard to the LF boundaries.

## 5. Simulation Results and Discussions

The addressed PV system generates a low voltage, and by using a boost converter, it is boosted to a suitable voltage, which appears at the DC-link. The capability of the proposed PV-DVR system is studied under different fault conditions. The effectiveness of the proposed, developed, and robust controllers for improving the performance of a PV system, which supplies AC loads and is linked to the grid of a new community in Egypt, as illustrated in Figure 1, is evaluated. The DVR is connected to the system, as shown in Figure 1. To keep its DC-link voltage constant, energy storage is used. The suggested green system is appropriate for balancing out the voltage in industrial buildings and small businesses. The ARO and GWO algorithms are implemented using the MATLAB global optimization toolbox on a computer with an Intel (R) Core (TM) i7-4700HQ CPU with a 2.40 GHz processing speed and 16.0 GB of memory. The optimal values for the PI controller’s gains, which correspond to the minimum objective function value, are obtained through the studied algorithms and are listed in Table 1. The studied system is evaluated in terms of improving the $V_{L}$ under two fault scenarios. The load side results are shown with and without the optimized DVR control system using the MATLAB/Simulink platform.

Case 1: Three-phase fault

**Figure 9:**
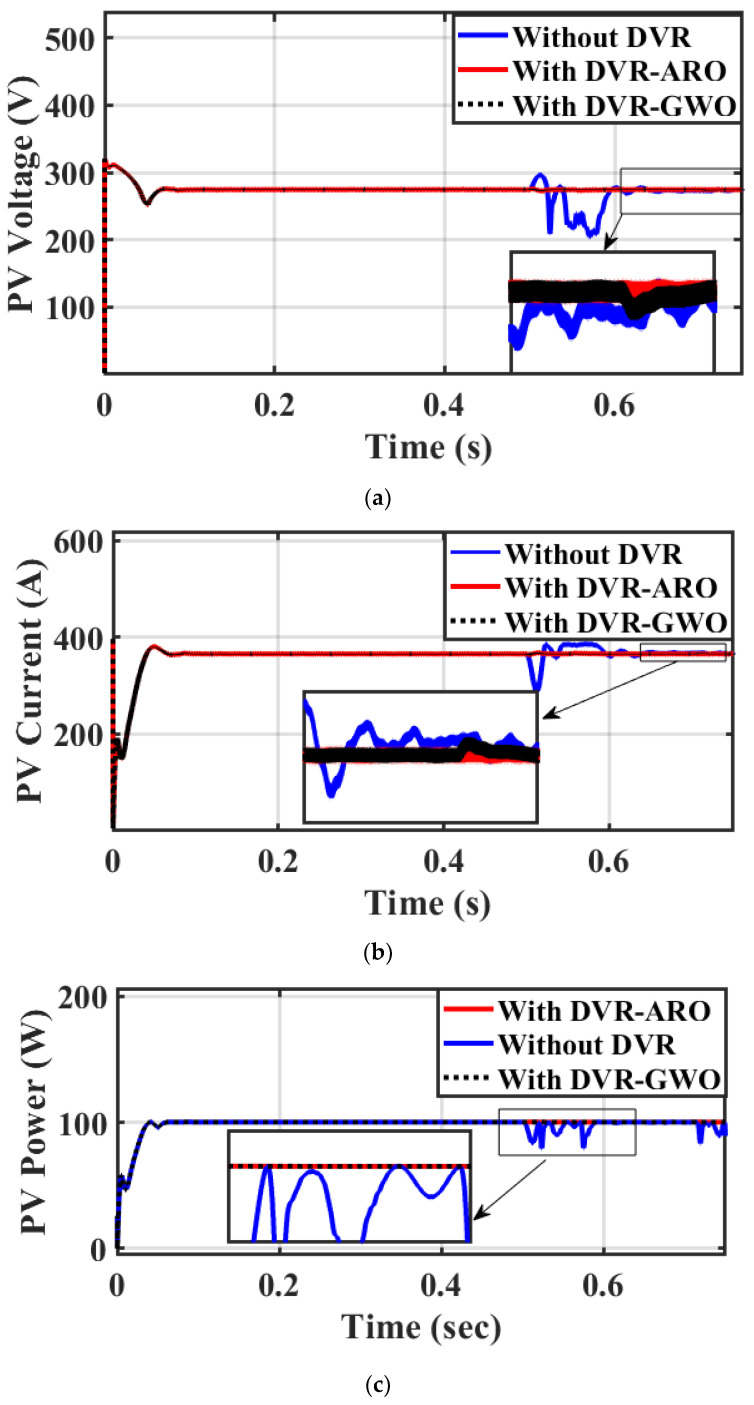
Simulation outcomes from PV without DVR, with GWO-PI for DVR, and ARO-PI for DVR under 3-phase fault: (**a**) voltage, (**b**) current, and (**c**) power.

A three-phase fault (worst condition) occurs at the PCC (F_2_), and the fault clearing time is expected to take place between 0.5 and 0.6 s. Figure 8 shows the response of the PV parameters (voltage, current, and power) in the case without a DVR (base case), a DVR with GWOs (C_1_), and a DVR with ARO (proposed C_2_). As a result of this fault, the voltage of the PV system drops, while the current increases to 1.2 p.u., causing a slight increase in the PV power waveforms, and this increase may disconnect the PV from the system. Figure 9 shows that using the DVR with the studied algorithms clears the fault in a short time (below 0.05 s). Furthermore, a lower settling time is achieved with the proposed C_2_, and it assures the role of the ARO technique. If a DVR is not integrated, the fault greatly impacts the system, causing voltage, current, and power waveform distortions. However, if a DVR is used, there is a noticeable improvement in the presented waveforms.

**Figure 10:**
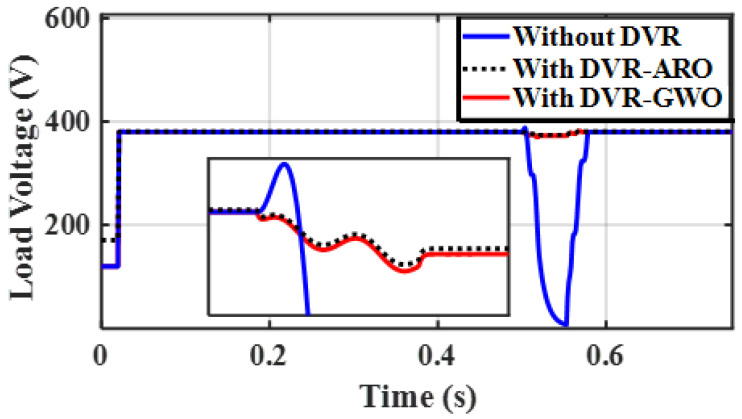
RMS of $V_{L}$ without DVR, with GWO-PI for DVR, and ARO-PI for DVR under the investigated fault.

**Figure 11:**
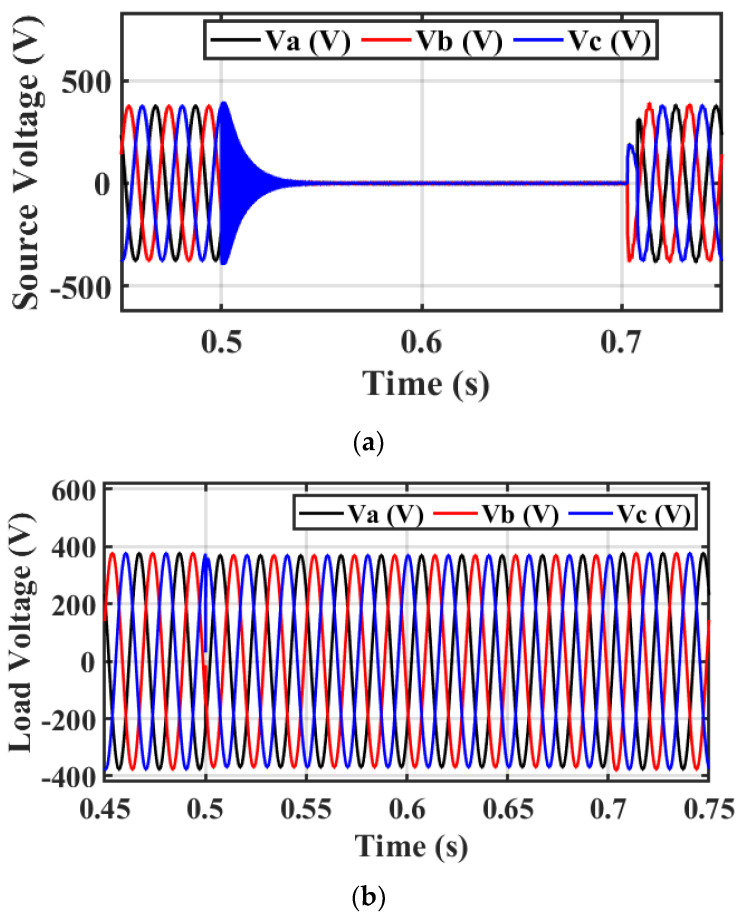

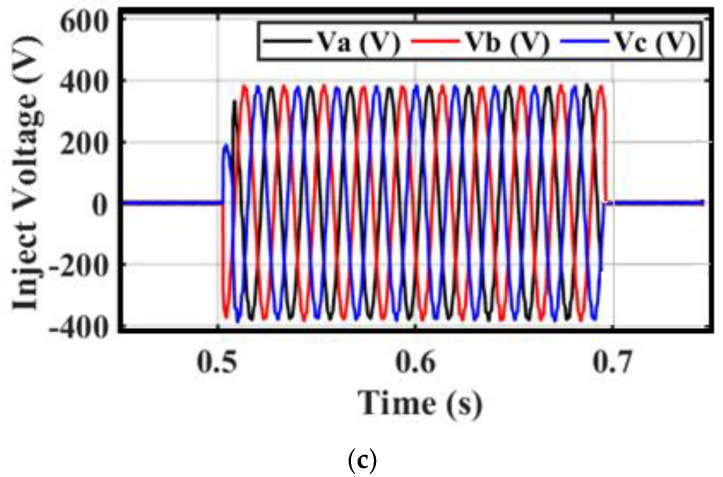
Simulation output under 3−phase fault of (**a**) source voltage, (**b**) $V_{L}$, and (**c**) injected/fed voltage.

Figure 10 demonstrates the role of the ARO-PI and GWO-PI controllers for the DVR. The optimized DVR can regulate the $V_{L}$ compared to the base case and minimize the presence of harmonics during fault conditions. Moreover, Figure 9 proves the superiority of the ARO technique compared to GWO. Figure 11a shows that the source voltage reaches a voltage of zero roughly during a fault period and returns to its steady state after fault clearance. Figure 11b shows the load voltage still operating at its rated value (1 p.u.) because of the suitable injected voltage value from the DVR, as seen in Figure 11c. Part (c) proves that the DVR performs well during a fault and ensures the continuous operation of the proposed system. It can be concluded that the DVR’s capability in injecting the needed voltage under the worst fault scenario assures the effectiveness of the DVR with its enhanced control system role.

All of the above-simulated results show the effectiveness of using the proposed ARO method for the DVR system in renewable systems for mitigating the negative effects of faults on the power system. Table 4 summarizes the performance index of the investigated system under the three operating scenarios.

Case 2: 25% voltage sag

**Figure 12:**
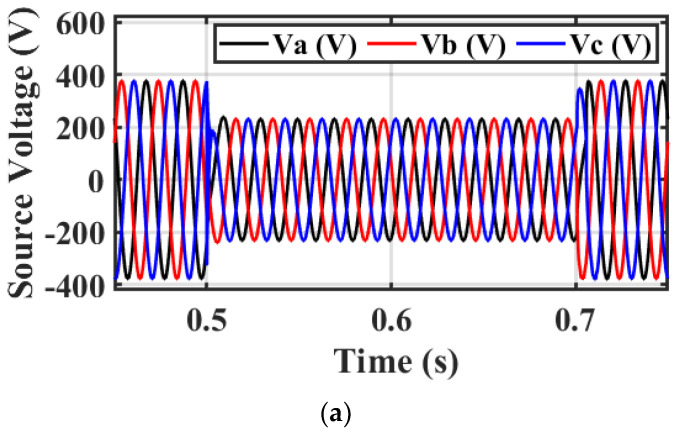

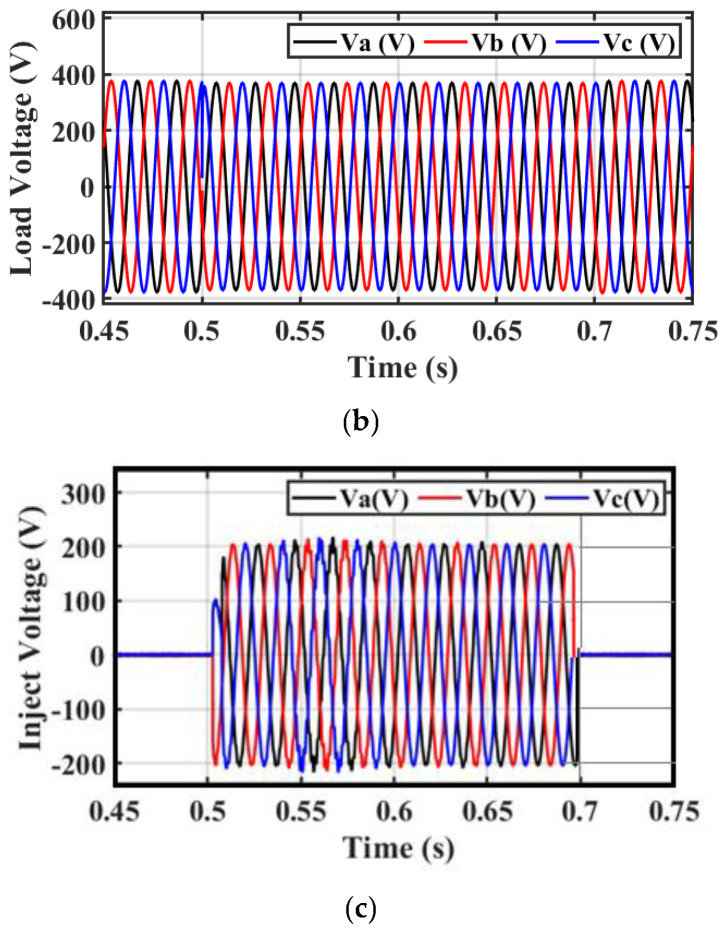
Simulation output under 25% $V_{S}$. (**a**) Source voltage, (**b**) $V_{L}$, and (**c**) injected/fed voltage with DVR−based investigated controller.

This scenario discusses testing the efficacy of the designed PI-ARO controller for DVR in improving the system performance under 25% $V_{S}$ with the condition between 0.5 and 0.7 s. Figure 12a–c depicts the source voltage, $V_{L}$, and the injected voltage from the DVR under the investigated voltage dip. The application of the DVR with its enhanced control system matches the $V_{L}$ with the source voltage by feeding the needed voltage. The injected voltage from the DVR is less than the injected voltage in case 1 because the source voltage fault is changed.

**Figure 13:**
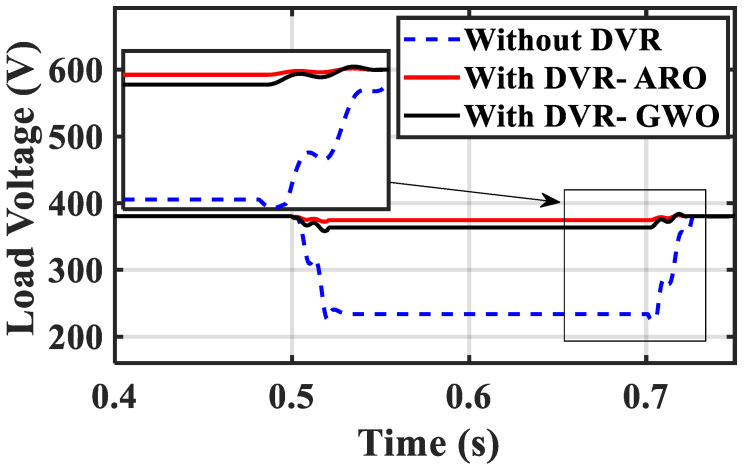
RMS of $V_{L}$ with and without optimized DVR during the fault period.

Figure 13 displays the RMS of $V_{L}$ with the three operating conditions under the studied fault period. The PI-ARO controller effectively reduced the maximum overshoot and achieved a rapid response. The PI−ARO approach is utilized to investigate the performance of the renewable power system’s grid under sagging test conditions with and without the DVR. Without the DVR, the $V_{L}$ drops to 215 V, while it remains at 377.5 V with the use of the DVR.

Case 3: %THD performance analysis

**Figure 14:**
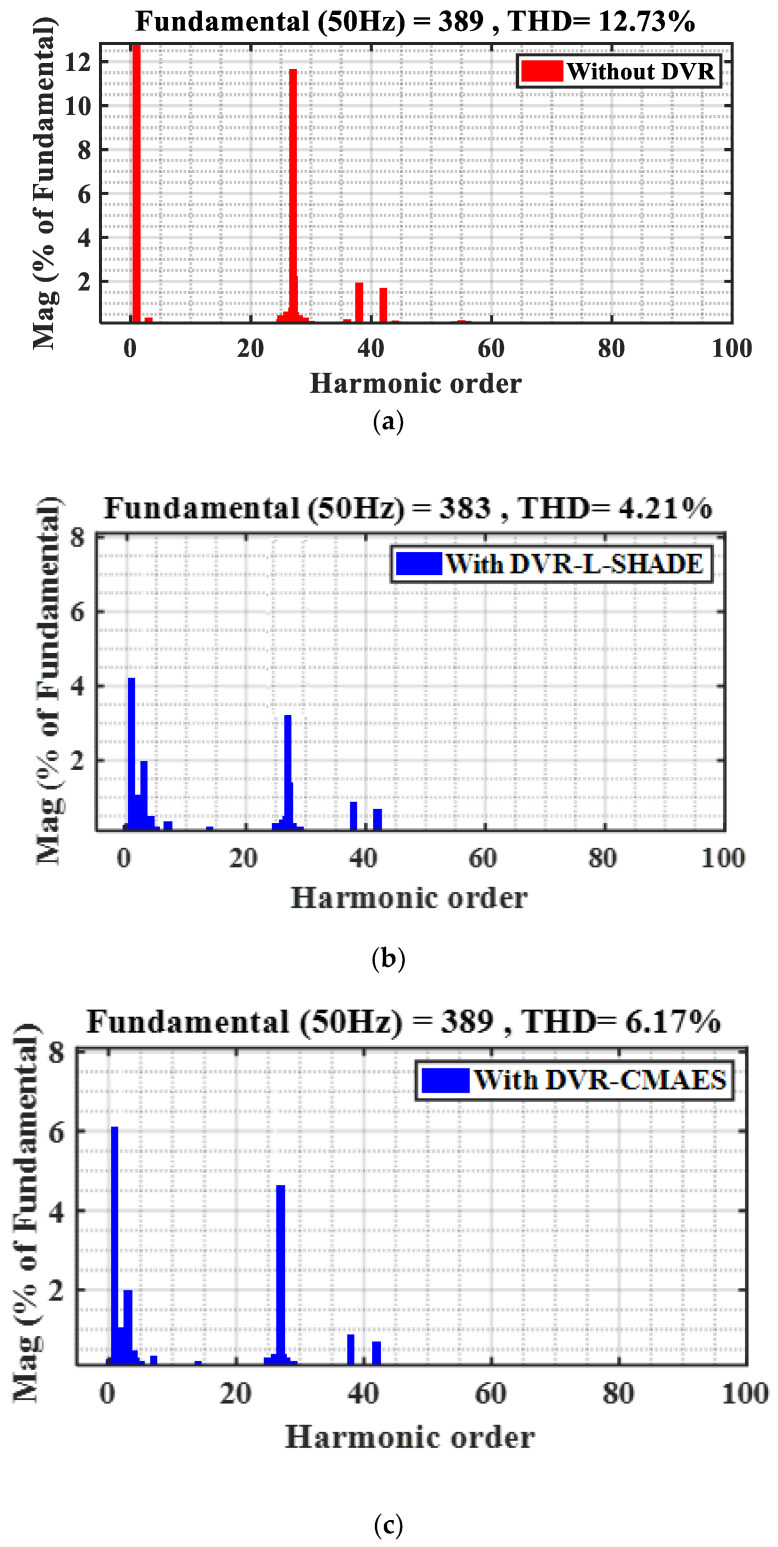

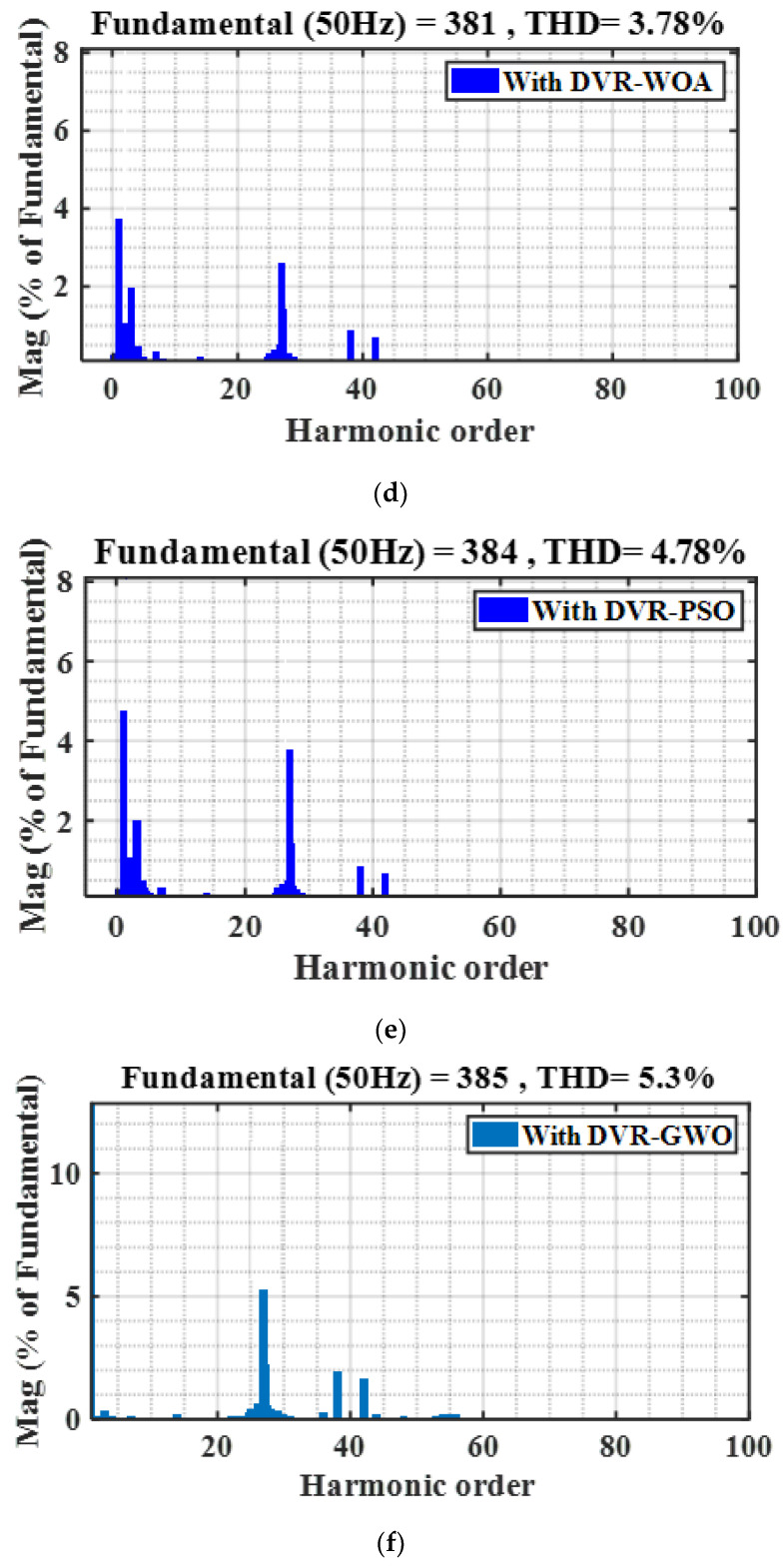

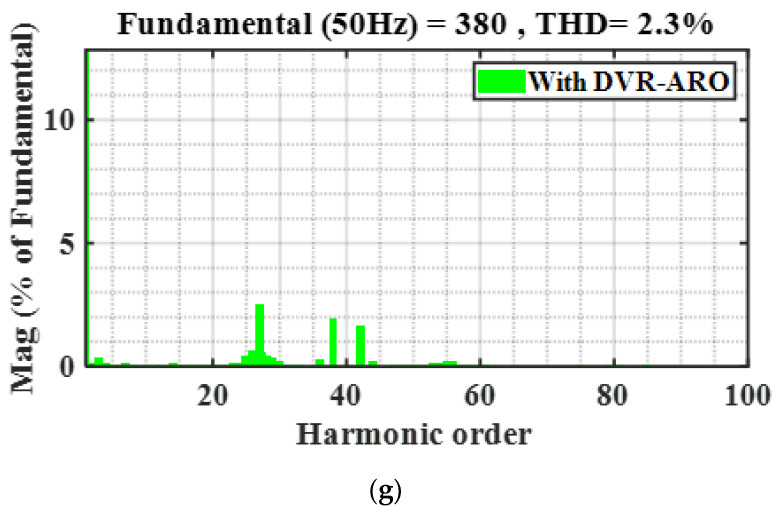
%THD values of $V_{L}$ under different optimizers. (**a**) Without optimized DVR. (**b**) With L−SHADE. (**c**) With CMAES. (**d**) With WOA. (**e**) With PSO. (**f**) With GWO. (**g**) With ARO (proposed).

The THD, which is being researched here to ensure the PQ restrictions, is one of the most crucial aspects. This subsection considers the L−SHADE, CMAES, WOA, PSO, GWO, and ARO algorithms both with and without the DVR system and performs an FFT analysis for the $V_{L}$ throughout the fault time. The THD is shown both with and without a DVR in the findings in Figure 14. According to Figure 14a–g, when the DVR is attached, the THD decreases significantly from 12.73% without the DVR to 2.3% with the DVR and ARO algorithm. It is a significant improvement in the $V_{L}$ waveforms that the THD was reduced by around 66.928%, 51.53%, 70.31%, 62.45%, 58.366%, and 81.9324% with L-SHADE, CMAES, WOA, PSO, GWO, and ARO, respectively.

Case 4: Relay response during three-phase fault

**Figure 15:**
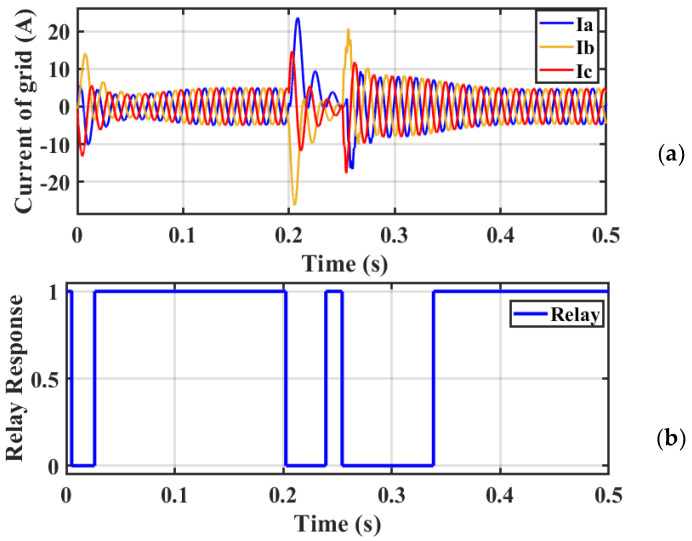
(**a**) Current waveform and (**b**) relay response during three−phase short circuit current without a trip.

Figure 15a,b demonstrates the simulation’s findings. Because of the excessive current that flows, whenever a malfunction (three-phase short circuit) happens, the circuit breaker (CB) is always turned off across the system. When a fault happens within 0.2 to 0.25 s, the relay might very well detect the current increase, but instead, trip immediately throughout the start-up; furthermore, after the failure has already been resolved, the current continues to remain large, and indeed, the relay causes excursions in the circuit.

**Figure 16:**
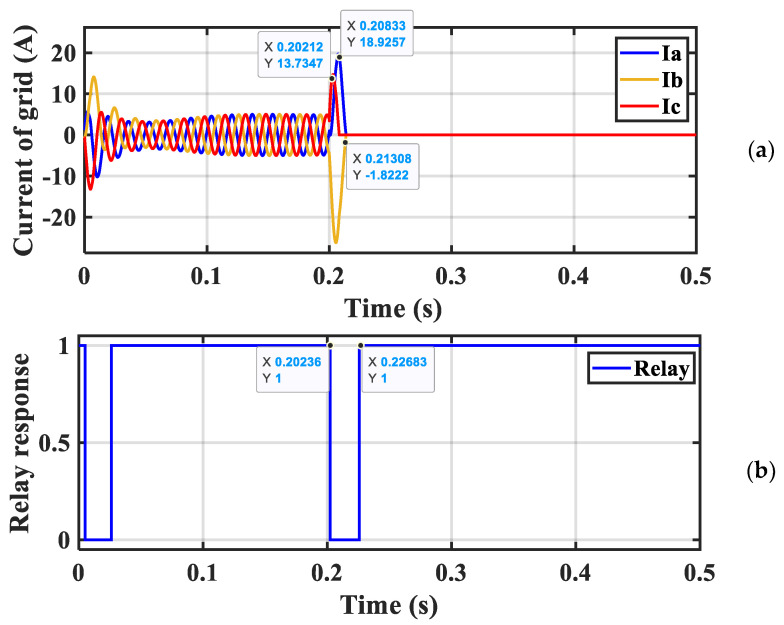
(**a**) Current waveform and (**b**) relay response during 3−phase fault with a trip.

In Figure 16a, it is clear that the grid current is constant and stable, but when a fault occurred (three-phase short circuit) at a time of 0.2 s, we found that the relay sensed a rise in the value of the current from 5A to 19A, the fault continued for 0.2 to 0.213 s, and the CB worked to separate the network current from the VSI. In Figure 16b, the response of the protection system against the surge current in the period from 0.2 to 0.22 s is noted, and the value changes from zero to one because it depends on digital electronic circuits, whose output is from zero to one. When the current increases to a high value from 5 A to 19 A, this can cause the DC-link capacitors to be damaged, and the system protection unit must intervene to separate the VSI from the grid.

## 6. Conclusions

To improve the functionality of a grid-tied PV system serving a new community in Egypt, this work presents an optimized DVR, which links the PCC and the load bus. The DVR controllers are designed and optimally tuned with six metaheuristics techniques (L-SHADE, CMAES, WOA, PSO, GWO, and ARO). The ARO-PI controller’s main goal is to support the control of the DVR voltage that is injected into the system to enhance the voltage profile under harsh operating cases. To identify the optimum PI controller settings, the ISE between the load and reference voltages is minimized using the studied techniques under the investigated scenarios (three-phase fault and voltage sags). Through the use of the studied cases, the system’s performance is evaluated. This work shows that the improved DVR control system with ARO reduces the distortions in the current, voltage, and power waveforms. Furthermore, operating the DVR with ARO improves the system’s transient response compared to the other methods in terms of less rise time, settling time, and overshoot. The proposed system leads to a significant improvement in the waveforms and system performance, including a continuous operation of the renewable energy source under faults. A short-circuit overcurrent protection system is also used to split the system and protect the power electronic circuits from high currents, ensuring that the PV can continue to function even under faults. These findings lead us to the conclusion that the suggested control system enhances the PQ for grid-connected PV systems.

Future research directions are as follows:

1. Developing a DVR controller with ARO for use in µGs to mitigate PQ problems.
2. Comparing the addition of FC with a battery and renewable systems at the DC side of the DVR to clarify the best structure and the merits and demerits of these structures.
3. Making use of brand-new hybrid optimization methods, where algorithms constructed using artificial intelligence may be crucial in dealing with the codependency of the control objectives and auto-tuning of the weighting variables.
4. The developed DVR can be utilized to realize the fault ride-through capability of PV and wind systems.
5. Making a comparison among new and old algorithms based on a statistical test such as the Wilcoxon signed-rank test to prove the role of the proposed one.

## Figures and Tables

**Table 1:** Comparison with previously published works.

Refs.	Publisher	Year	Applied Controller	System Simplicity	THD Analysis	Stability Analysis (SA)	Studied Events	Program	Remarks
[40]	IEEE	2020	PI	✗	✗	✗	Stable and unstable $V_{S}$	PSCAD/EMTDC	Safe grid linked (PV-WT) from $V_{F}$ with SMES and battery-based DVR.
[41]	MDPI	2023	FCS-MPC	✗	✓	✗	Voltage dips and swells	Matlab/Simulink and dSpace 1103 controller board	Compensated voltage under grid $V_{S}$/swell scenarios to save the load voltage stable.
[42]	IEEE	2020	PI	✗	✗	✗	$V_{S}$ and phase jumps (PJs)	MATLAB/Simulink and HIL	Maximized sensitive loads (SLs) and VQ with the most effective DVR usage.
[43]	MDPI	2021	PI	✗	✗	✗	$V_{S}$	MATLAB/Simulink	To address the DVR capacity issue (limited by restorative energy), an innovative DVR architecture (two 3-phase input matrix converters without a capacitor in the DC-link side (DCLS)) was implemented.
[44]	IEEE	2019	Hysteresis	✗	✗	✗	Wide range of $V_{S}$	MATLAB/Simulink	Enhanced VQ in using SFCL and DVR via SMES Realized the entanglement of the immediate magnitudes and phase angles of the line voltages, and decreased the system’s price.
[45]	IEEE	2019	PI	✓	✓	✗	Balanced and unbalanced $V_{S}$	PSCAD/EMTDC	Positively functioned during short-circuit fault current limiting and compensation for $V_{F}$ regimes.
[46]	Elsevier	2020	Fuzzy type 1, 2, and PI	✗	✗	✗	$V_{S}$ and $V_{W}$	MATLAB/Simulink and HIL	Voltage correction was carried out without the need for a PLL circuit or an extensive DC capacitor.
[47]	Elsevier	2019	PI	✓	✗	✗	$V_{S}$, $V_{W}$ and unbalanced V	Homer software	Improved the dynamic performance of a hybrid power system (FC-WT-PV battery). It was advantageous for developing communities and SCs.
Current study			ARO-PI	✓	✓	✓	%THD performance analysis, relay response during 3-phase faults, 25% voltage sag, and 3-phase fault.	MATLAB/Simulink	Improving the DVR control system with ARO-PI to enhance PQ under severe events with low THD. Protection and relay over current is studied.

**Table 2:** The system’s components.

Parameters	Value and Unit
PV array’s peak power level voltage $\left(V_{PV}\right)$	273.5 V
Open circuit voltage of one PV module $\left(V_{OC}\right)$	64.2 V
Networking voltage	11 kV
Networking-based frequency	314 rad\s
Inverter carrier frequency	2 kHz
Sample period	1 µs
The boost’s inductor	5 µH
DC-link reference voltage	250 V
DC-link measured voltage	250 V
DC-connection capacitor	24 mF

**Table 3:** The optimized parameters of the PI controllers.

Algorithms	d-Axis		q-Axis	
	$K_{pd}$	$K_{id}$	$K_{pq}$	$K_{iq}$
GWO	8.5420	199.0017	77.3220	190.6024
L-SHADE	1.6952	0.2057	9.0366	3.2531
CMAES	1.7304	0.2062	9.0374	3.4842
WOA	1.6675	0.2056	9.0374	3.1668
PSO	1.7248	0.2053	9.0454	3.3255
ARO (proposed)	1.8751	2.9975	2.9791	0.7297

**Table 4:** Comparative analysis of a three-phase fault under the three operating conditions.

Parameters	Ref. Values	Operating Conditions under the Investigated Fault						
		Without DVR	DVR-Based PI-GWO	DVR-Based PI-L-SHADE	DVR-Based PI-CMAES	DVR-Based PI-WOA	DVR-Based PI-PSO	DVR with PI-ARO (Proposed)
PV voltage	275 V	210.2 V	273.3 V	273.8 V	272.5 V	273.9 V	273.5 V	274.2 V
PV current	362 A	385 A	366 A	364.2 A	364.8 A	365.07 A	365 A	365.12 A
PV power	100 kW	83.4 kW	99.87 kW	99.87 kW	99.83 kW	99.89 kW	99.83 kW	99.92 kW
Load voltage	380 V	Zero V	375.12 V	375.82 V	375.92 V	375.32 V	375.47 V	376.4 V

## Data Availability

Data are available upon request from the authors.

## Abbreviations

DVR: Dynamic voltage resistor	THD: Total harmonic distortion
UP: Urban population	PV: Solar photovoltaic
$V_{L}:$ Load voltage	$V_{S}:$ Voltage sag
SCs: Sustainable cities	FCs: Fuel cells
GDP: Gross domestic product	PCC: Point of common coupling
L-SHADE: Linear population size reduction	PQ: Power quality
pu: Per unit	DVSI: Dual voltage source inverter
µGs: Microgrids	STC: Static transfer switch
ARO: Artificial Rabbits Optimization	DF: Detour foraging
WOM: Whale optimization method	PLL: Phase-locked loop
CB: Circuit breaker	ISE: Integral square error
CMAES: Covariance matrix adaptation evolution strategy	FFs: Fossil fuels
